# Knowledge, attitudes, and practices of patients with multiple myeloma regarding venous thromboembolism: a cross-sectional study

**DOI:** 10.3389/fcvm.2026.1722955

**Published:** 2026-04-24

**Authors:** Rui Zhang, Yue Weng, Shuting Wu, Ting Liao, Ping Pan, Hongmei Gou, Wei Ding, Mingjiang Dong, Zhongwen Yang

**Affiliations:** Hematological Department, Bazhong Central Hospital, Bazhong, Sichuan, China

**Keywords:** cross-sectional study, knowledge, attitudes, practice, multiple myeloma, patient education, preventive health services, venous thromboembolism

## Abstract

**Introduction:**

Venous thromboembolism (VTE) is a significant complication in multiple myeloma (MM) patients, yet patient awareness and preventive behaviors in this population have received limited research attention.

**Methods:**

This cross-sectional survey, conducted at Bazhong Central Hospital (January-September 2024), assessed the knowledge, attitudes, and practices (KAP) of 504 MM patients regarding VTE.

**Results:**

Participants were predominantly male (65.1%), with 57.7% reporting prior VTE. Mean scores (SD) were suboptimal: knowledge (8.97 ± 2.92, range 0–13), attitude (29.59 ± 2.70, range 14–70), and practice (44.03 ± 4.07, range 10–50). Positive correlations were found between knowledge-attitude (*r* = 0.141, *P* = 0.002), knowledge-practice (*r* = 0.281, *P* < 0.001), and attitude-practice (*r* = 0.159, *P* < 0.001). Structural equation modeling revealed knowledge directly influenced attitude (*β* = 0.761, *P* < 0.001), attitude directly affected practice (*β* = 0.806, *P* < 0.001), and knowledge indirectly impacted practice via attitude (*β* = 0.613, *P* < 0.001).

**Discussion:**

Findings highlight gaps in VTE knowledge and negative attitudes among MM patients, despite proactive practices. Improving patient education on VTE may enhance attitudes and behaviors, potentially reducing VTE risk in this high-risk population. Targeted interventions are warranted to optimize VTE prevention strategies.

## Introduction

Multiple myeloma (MM) is a malignancy of plasma cells, which are integral components of the immune system responsible for antibody production. In MM, plasma cells proliferate abnormally and cluster in the bone marrow, producing excessive antibodies that frequently lead to organ damage, including kidney injury, hypercalcemia, anemia, and bone lesions (1). Despite advancements in treatment options, the global incidence of MM has steadily increased over the past few decades, and it now accounts for approximately 17% of all hematologic malignancies (2).

Patients with MM face a significantly elevated risk of venous thromboembolism (VTE), estimated to be nine times higher than that of the general population (3, 4). This heightened risk is multifactorial, driven by the hypercoagulable state induced by MM, as well as treatment-related factors such as the use of immunomodulators and chemotherapy. Remarkably, even during the asymptomatic monoclonal gammopathy of undetermined significance (MGUS) stage, the risk of VTE is notably increased (5–8). Most VTE events in MM patients occur within the first six months of initiating treatment, underscoring the critical importance of early thrombotic risk assessment and prophylactic strategies for all patients beginning anti-myeloma therapy (3, 9, 10). Recommended prophylactic measures include low-molecular-weight heparin, warfarin, and direct oral anticoagulants (11). However, despite these established guidelines, patient adherence to VTE prevention strategies remains suboptimal across various cancer populations. Unlike many solid tumors, where thrombosis risk is often primarily associated with tumor burden or advanced disease stage, MM presents a uniquely treatment-driven thrombotic profile. The widespread use of immunomodulatory agents such as thalidomide and lenalidomide, particularly when combined with high-dose dexamethasone, substantially amplifies VTE risk and necessitates individualized prophylactic strategies. Moreover, MM is characterized by a chronic, relapsing disease trajectory, requiring prolonged therapy and repeated risk reassessment, which places greater demands on patient understanding and sustained adherence to preventive measures. Compared with other hematologic malignancies, the thrombotic risk in MM is more tightly interwoven with therapeutic regimens rather than disease biology alone, making patient awareness and engagement particularly critical. Therefore, MM patients represent a distinct and clinically meaningful population for investigating VTE-related knowledge, attitudes, and practices. Previous studies in other malignancies have demonstrated significant knowledge gaps and negative attitudes toward anticoagulation therapy, which directly impact preventive behaviors and clinical outcomes (12, 13). To date, limited research has specifically examined patient awareness and behaviors regarding VTE in the MM population, representing a critical gap in understanding how to optimize prevention strategies in this high-risk group.

The Knowledge, Attitude, and Practices (KAP) survey serves as a valuable diagnostic research tool to assess an individual's understanding, beliefs, and behaviors related to a specific topic. Within the context of health literacy, the KAP framework operates on the premise that knowledge positively shapes attitudes, which in turn influence behaviors (14, 15). In the case of MM patients, evaluating their KAP regarding VTE is particularly important because effective thromboprophylaxis requires not only the selection of appropriate anticoagulants but also an understanding of the patient's preferences, adherence likelihood, and comprehension of the disease. This ensures that the most minimally invasive and cost-effective strategies can be tailored to account for individual factors such as age, frailty, and economic considerations (16).

Addressing this gap is clinically significant, as inadequate knowledge or negative attitudes may hinder adherence to preventive measures, ultimately increasing morbidity and mortality. Understanding KAP gaps in this high-risk population allows for the development of targeted interventions to improve VTE prevention and management. Such efforts have the potential to reduce complications and enhance patient outcomes. Therefore, this study aimed to evaluate the KAP of MM patients regarding VTE to inform future individualized prevention strategies.

## Material and methods

### Study design and participants

This cross-sectional study was conducted at Bazhong Central Hospital from January to September, 2024, focusing on patients with multiple myeloma (MM). A convenience sampling strategy was employed, recruiting consecutive MM patients who met the eligibility criteria during the study period. Ethical approval was obtained from the Bazhong Central Hospital Ethics Committee, and informed consent was secured from all participants.

Eligible participants were consecutive patients receiving treatment for multiple myeloma, including those with newly diagnosed and relapsed/refractory disease. For elderly patients who were unable to independently comprehend the questionnaire, responses were recorded with the assistance of family members. Patients were excluded if they had severe cognitive impairment that prevented questionnaire completion even with assistance, refused to provide informed consent, or submitted incomplete questionnaire responses (with >20% missing data).

### Questionnaire Introduction

The questionnaire was developed based on guidelines and relevant literature (17–19), with additional insights drawn from more than two subsequent studies. Following its initial design, the questionnaire was reviewed and revised based on feedback from three hematology experts to ensure content validity. A small-scale pilot test involving 30 participants was conducted, resulting in a Cronbach's α coefficient of 0.861, which indicated good reliability. To further evaluate construct validity, confirmatory factor analysis (CFA) was performed to assess the three-factor structure corresponding to the knowledge, attitude, and practice dimensions. The model demonstrated good fit, with a root mean square error of approximation (RMSEA) of 0.046, standardized root mean square residual (SRMR) of 0.055, Tucker–Lewis index (TLI) of 0.855, and comparative fit index (CFI) of 0.865, all meeting commonly accepted criteria for adequate fit (Supplementary Table S1 and Supplementary Figure S1). These findings support the structural validity of the questionnaire. For patients reporting a history of VTE, medical records were reviewed to confirm the diagnosis and obtain clinical details. All VTE events were verified through review of imaging studies, discharge summaries, or physician documentation to ensure accuracy of self-reported data. In this study, prior VTE history included any documented venous thromboembolic event occurring before or after MM diagnosis, including deep vein thrombosis, pulmonary embolism, catheter-related thrombosis, and other clinically confirmed venous thrombotic events. Both recent and remote historical events were recorded if documented in medical records. The final version of the questionnaire, written in Chinese, consisted of four dimensions with a total of 55 items: 18 items for basic information, 13 for knowledge, 14 for attitudes, and 10 for practices. For statistical analysis, scores were assigned according to the nature of each item. In the knowledge dimension, correct answers were awarded 1 point, while incorrect or unclear answers received 0 points, with a total possible score ranging from 0 to 13. The attitude dimension included both positive and negative items; positive items (A1–A3, A5–A6, A12–A14) were scored from 5 (strongly agree) to 1 (strongly disagree), while negative items (A4, A7–A11) were reverse-scored, yielding a total score range of 14–70. The practice dimension consisted entirely of positive items, scored from 5 (always) to 1 (never), with a total score range of 10–50. To define adequate knowledge, positive attitudes, and proactive practices, a scoring threshold of >70% was established, based on prior research (20). This threshold was selected based on established benchmarks in health literacy research and has been validated in similar KAP studies across various chronic disease populations. The 70% cutoff represents a balance between achievable competency and meaningful health behavior change.

The questionnaire was distributed through both consultation rooms and WeChat groups, using a QR code generated through Wenjuanxing. Bazhong Central Hospital served as the central hub for survey distribution.

### Sample size calculation

The sample size was calculated using the standard formula for cross-sectional studies: *n* = (Z₁−*α*/2/*δ*)² × *p* × (1−p), where *α* = 0.05 (Z₁−*α*/2 = 1.96), *δ* = 0.05, and *p* = 0.5 to maximize the sample size. The minimum required sample size was calculated as 384. Assuming a response rate of 80%, the final target sample size was set at 480 participants.

### Statistical methods

Data were analyzed using R version 4.3.2 and Stata version 18.0 (StataCorp, College Station, TX, USA). Continuous variables were expressed as mean ± standard deviation (SD), and categorical variables were presented as number (percentage). Group comparisons of KAP scores across demographic and clinical characteristics were performed using independent-samples t-tests or one-way analysis of variance (ANOVA). Spearman's rank correlation analysis was conducted to assess the relationships among knowledge, attitude, and practice scores. For logistic regression analysis, KAP scores were dichotomized according to the median value of each dimension. Univariable logistic regression analyses were first conducted, and variables with *P* < 0.05 were subsequently entered into multivariable logistic regression models to identify independent factors associated with knowledge, attitude, and practice levels. Prior to multivariable logistic regression, multicollinearity was assessed using variance inflation factors (VIFs). For categorical variables, generalized VIFs (GVIFs) were calculated and adjusted as GVIF^[1/(2 × Df)]. All adjusted values were <2, indicating no significant multicollinearity. Therefore, all covariates were retained in the final models. Based on the KAP framework, structural equation modeling (SEM) was performed to examine the direct and indirect relationships among knowledge, attitude, and practice. SEM analysis was conducted to test the hypotheses that (H1) knowledge directly affects attitude, (H2) knowledge directly affects practice, and (H3) knowledge indirectly affects practice through attitude. Model fit was evaluated using the root mean square error of approximation (RMSEA), standardized root mean square residual (SRMR), Tucker–Lewis index (TLI), and comparative fit index (CFI). All statistical tests were two-sided, and *P* < 0.05 was considered statistically significant.

## Results

Initially, 508 questionnaires were collected. The following were excluded: one case where age was erroneously recorded as >150 years, two cases with abnormal height and weight, and one case where the Padua score was marked as “unknown”. The final dataset included 504 valid responses. The internal consistency of the questionnaire was robust overall and across sections. The overall reliability (Cronbach's *α*) was 0.8784, with section-specific scores of 0.8123 for knowledge, 0.7679 for attitude, and 0.7363 for practice. The overall validity (Kaiser-Meyer-Olkin, KMO value) was 0.8982.

### Demographic information on participants

Of the 504 participants, 328 (65.1%) were male, 214 (42.5%) were aged 70 years or older, and 367 (72.8%) had a BMI within the normal range. Additionally, 291 (57.7%) had experienced venous thromboembolism, including both recent and historical events occurring before or after MM diagnosis. 309 (61.3%) had IgG myeloma subtype, 288 (57.1%) were in stage II, 452 (89.7%) had a Padua score of 0–3 (low risk), 393 (78.0%) had a Khorana score of 1–2 (medium risk), and 300 (59.5%) had an IMPEDE Venous Thromboembolism Risk Assessment Model (IMPEDEVTE) score of 4–7 (medium risk). The mean (SD) scores for knowledge, attitude, and practice were 8.97 (2.92), 29.59 (2.70), and 44.03 (4.07), respectively. Knowledge scores differed significantly based on gender (*P* = 0.023), age (*P* < 0.013), residence (*P* < 0.001), education level (*P* < 0.001), employment status (*P* < 0.001), monthly income (*P* < 0.001), marital status (*P* = 0.009), health insurance type (*P* < 0.001), risk disclosure (*P* < 0.001), Padua score (*P* < 0.001), Khorana score (*P* = 0.001), and IMPEDEVTE score (*P* = 0.007). Attitude scores varied significantly by residence (*P* = 0.042), education level (*P* = 0.010), employment status (*P* < 0.001), monthly income (*P* = 0.027), risk disclosure (*P* = 0.003), venous thromboembolism history (*P* < 0.001), multiple myeloma type (*P* = 0.035), Padua score (*P* < 0.001), and Khorana score (*P* = 0.001). Practice scores showed significant variation by residence (*P* = 0.003), education level (*P* < 0.001), employment status (*P* < 0.001), monthly income (*P* < 0.001), health insurance type (*P* = 0.006), risk disclosure (*P* < 0.001), Padua score (*P* < 0.001), Khorana score (*P* = 0.001), and IMPEDEVTE score (*P* = 0.001) (Table 1).

**Table 1:** Baseline characteristics and KAP score comparisons across demographic and clinical variables (*N* = 504).

*N* = 504	*N*(%)	Knowledge	*P*	Attitude	*P*	Practice	*P*
		mean (SD)		mean (SD)		mean (SD)	
Total score (ranges)	504 (100.0)	8.97 (2.92) [0–13]		29.59 (2.70) [14–70]		44.03 (4.07) [10–50]	
Gender			0.023		0.094		0.093
Male	328 (65.1)	9.19 (2.79)		29.72 (2.59)		44.30 (3.78)	
Female	176 (34.9)	8.56 (3.11)		29.35 (2.88)		43.53 (4.53)	
Age			0.013		0.133		0.070
Under 60 years old	109 (21.6)	9.27 (3.32)		29.73 (2.84)		44.70 (4.18)	
60–69 years old	181 (35.9)	8.86 (3.16)		29.85 (2.64)		43.66 (3.95)	
70 years old and above	214 (42.5)	8.91 (2.46)		29.30 (2.65)		44.01 (4.08)	
BMI			0.106		0.071		0.134
<18.5	29 (5.8)	8.21 (3.35)		30.07 (3.45)		42.48 (4.72)	
18.49–23.99	367 (72.8)	9.05 (2.59)		29.46 (2.71)		44.17 (3.99)	
> = 24.0	108 (21.4)	8.88 (3.74)		29.93 (2.40)		43.98 (4.11)	
Ethnicity			0.250		0.439		0.124
Han	489 (97.0)	8.94 (2.94)		29.60 (2.66)		44.06 (4.11)	
Minority	15 (3.0)	9.87 (2.29)		29.40 (3.92)		43.07 (2.40)	
Residence			<0.001		0.042		0.003
Rural/suburban	280 (55.6)	8.47 (2.97)		29.38 (2.73)		43.50 (4.24)	
Urban	224 (44.4)	9.58 (2.74)		29.86 (2.63)		44.70 (3.74)	
Education			<0.001		0.010		<0.001
Middle school or below	220 (43.7)	8.23 (3.29)		29.50 (2.92)		43.00 (4.69)	
High school/technical school	234 (46.4)	9.41 (2.21)		29.44 (2.39)		44.70 (3.13)	
Associate degree/bachelor's degree	50 (9.9)	10.12 (3.32)		30.68 (2.83)		45.44 (3.98)	
Employment status			<0.001		<0.001		<0.001
Full-time	109 (21.6)	9.89 (2.20)		30.04 (2.47)		45.15 (3.25)	
Part-time/self-employed/freelancer	208 (41.3)	9.46 (2.14)		29.31 (2.37)		44.72 (3.29)	
Unemployed/laid off	48 (9.5)	5.02 (4.49)		31.65 (3.66)		38.50 (4.65)	
Full-time homemaker	89 (17.7)	9.07 (2.14)		28.52 (2.32)		44.17 (3.98)	
Retired	50 (9.9)	8.50 (3.41)		29.74 (2.83)		43.84 (4.11)	
Monthly income			<0.001		0.027		<0.001
<2,000	32 (6.3)	5.59 (4.05)		29.47 (3.62)		39.81 (4.54)	
2,000–5,000	432 (85.7)	9.07 (2.67)		29.49 (2.57)		44.29 (3.84)	
>5,000	40 (7.9)	10.50 (2.51)		30.75 (2.96)		44.65 (4.28)	
Marital status			0.009		0.368		0.237
Married	471 (93.5)	9.04 (2.90)		29.61 (2.66)		44.10 (4.04)	
Divorced	33 (6.5)	7.85 (3.06)		29.27 (3.16)		43.12 (4.46)	
Type of health insurance			<0.001		0.354		0.006
Social medical insurance only	425 (84.3)	8.83 (2.85)		29.54 (2.72)		43.88 (3.98)	
Both social and commercial medical insurance	64 (12.7)	10.09 (3.25)		29.98 (2.52)		45.39 (4.29)	
No insurance	15 (3.0)	8.13 (2.59)		29.27 (2.71)		42.67 (4.43)	
Whether the doctor informed you about the risk of venous thromboembolism			<0.001		0.003		<0.001
No	24 (4.8)	1.50 (3.44)		31.42 (3.37)		36.75 (3.31)	
Yes	480 (95.2)	9.34 (2.34)		29.50 (2.63)		44.40 (3.75)	
Venous thromboembolism			0.435		<0.001		0.530
No	213 (42.3)	8.43 (3.77)		30.25 (2.76)		43.86 (4.32)	
Yes	291 (57.7)	9.36 (2.00)		29.11 (2.55)		44.16 (3.87)	
Type of multiple myeloma			0.933		0.035		0.510
IgA myeloma	128 (25.4)	8.87 (3.12)		29.52 (2.63)		43.91 (4.22)	
IgG myeloma	309 (61.3)	9.01 (2.74)		29.49 (2.64)		44.10 (4.01)	
Light chain myeloma	55 (10.9)	9.04 (3.15)		29.71 (2.58)		44.33 (3.90)	
Other (b/d/e/g/h)	12 (2.4)	8.50 (4.19)		32.58 (3.85)		42.25 (4.61)	
Multiple Myeloma Staging (R-ISS)			0.712		0.153		0.989
Stage I	84 (16.7)	9.18 (2.38)		30.04 (2.68)		44.29 (3.56)	
Stage II	288 (57.1)	8.91 (2.89)		29.41 (2.61)		44.11 (3.79)	
Stage III	132 (26.2)	8.95 (3.29)		29.70 (2.88)		43.70 (4.89)	
Padua Score			<0.001		<0.001		<0.001
Low risk = 0–3 points	452 (89.7)	9.44 (2.29)		29.30 (2.44)		44.67 (3.54)	
High risk ≥ 4 points	52 (10.3)	4.88 (4.34)		32.10 (3.47)		38.52 (4.21)	
Khorana Score			0.001		<0.001		<0.001
Low risk: total score 0 point	95 (18.8)	7.25 (4.23)		31.00 (3.26)		41.89 (4.70)	
Medium risk: total socre 1–2 points	393 (78.0)	9.35 (2.37)		29.30 (2.45)		44.58 (3.72)	
High risk: total score > 3 points	16 (3.2)	9.69 (2.15)		28.38 (2.00)		43.44 (4.08)	
IMPEDEVTE Score			0.007		0.197		0.001
Low risk: total score ≤ 3 points	191 (37.9)	8.24 (3.66)		29.86 (2.72)		43.35 (4.20)	
Medium risk: total score 4–7 points	300 (59.5)	9.45 (2.24)		29.44 (2.65)		44.62 (3.70)	
High risk: total score ≥ 8 points	13 (2.6)	8.38 (2.53)		29.15 (3.31)		40.62 (6.65)	

KAP, knowledge–attitude–practice; BMI, body mass index; R-ISS, revised international staging system; IMPEDE VTE, IMPEDE venous thromboembolism risk assessment model.

### Distribution of responses to knowledge, attitude, and practice

The distribution of knowledge dimensions showed that the questions with the highest number of participants choosing the “Not sure” option were “Multiple myeloma patients only need to prevent venous thromboembolism within the first six months of treatment”. (K12) with 36.3% and “If a multiple myeloma patient develops venous thromboembolism, they need to discontinue treatment for multiple myeloma” (K10) with 30.4%. Also, the largest percentage of people (64.3%) chose the wrong answer for K10 (the correct answer was “False”), demonstrating that the participants' knowledge in the above areas needs to be improved urgently (Table 2.1). When it comes to related attitudes, 58.7% were very concerned about the cost of treatment for venous thromboembolism (A7), 61.1% were very worried about the risk of developing venous thrombosis (A8), 63.7% were already reported treatment-related fatigue (A9), and 60.5% stated that the prevention of venous thromboembolism would be burdensome (A10) (Table 2.2). Responses to the practice dimension showed that the majority of participants had active practices (choosing either “Strongly agree” or “Agree”). However, 10.9% were also neutral about being proactive in learning about venous thromboembolism (P1) and 10.3% were neutral about sharing their experience and knowledge of venous thrombosis risk management with other patients (P8) (Table 2.3).

**Table 2.1:** Distribution of responses to knowledge dimension questions regarding VTE in MM patients.

Knowledge	True	False	Not sure
1. Patients with multiple myeloma are prone to developing venous thromboembolism. T	468 (92.9%)	1 (0.2%)	35 (6.9%)
2. Older patients with multiple myeloma are more likely to develop venous thromboembolism. T	456 (90.5%)	1 (0.2%)	47 (9.3%)
3. Patients with a history of venous thromboembolism are more likely to develop it again. T	413 (81.9%)	2 (0.4%)	89 (17.7%)
4. Patients with a recent history of surgery are more likely to develop venous thromboembolism. T	349 (69.2%)	9 (1.8%)	146 (29%)
5. Venous thromboembolism is likely to occur within six months after treatment for multiple myeloma or during a relapse. T	346 (68.7%)	9 (1.8%)	149 (29.6%)
6. Treatment-related factors for multiple myeloma significantly increase the incidence of venous thromboembolism. T	363 (72%)	7 (1.4%)	134 (26.6%)
7. Patients with multiple myeloma combined with other underlying conditions (e.g., obesity, diabetes, myocardial infarction) are more likely to develop venous thromboembolism. T	406 (80.6%)	3 (0.6%)	95 (18.8%)
8. Patients with multiple myeloma who have minimal physical activity are more likely to develop venous thromboembolism. T	389 (77.2%)	10 (2%)	105 (20.8%)
9. Patients with multiple myeloma undergoing long-term combined treatment with immunomodulators, multi-drug chemotherapy, or glucocorticoids require continuous medication to prevent venous thromboembolism. T	419 (83.1%)	3 (0.6%)	82 (16.3%)
10. If a multiple myeloma patient develops venous thromboembolism, they need to discontinue treatment for multiple myelom F	324 (64.3%)	27 (5.4%)	153 (30.4%)
11. Long-term anticoagulant therapy is not needed for multiple myeloma patients with recurrent venous thromboembolism. F	39 (7.7%)	373 (74%)	92 (18.3%)
12. Multiple myeloma patients only need to prevent venous thromboembolism within the first six months of treatment. F	112 (22.2%)	209 (41.5%)	183 (36.3%)
13. Patients with a recent history of surgery are less likely to develop venous thromboembolism. F	54(10.7%)	301(59.7%)	149(29.6%)

**Table 2.2:** Distribution of responses to attitude dimension questions regarding VTE prevention and management.

Attitude	Strongly agree	Agree	Neutral	Disagree	Strongly disagree
1. In my opinion, venous thrombosis has a significant impact on my health. (P)	409 (81.2%)	61 (12.1%)	27 (5.4%)	7 (1.4%)	0 (0%)
2. I believe it is important to take preventive measures to reduce the risk of venous thrombosis. (P)	350 (69.4%)	120 (23.8%)	27 (5.4%)	7 (1.4%)	0 (0%)
3. I think it is very important to understand the risk factors and prevention methods for venous thrombosis. (P)	344 (68.3%)	124 (24.6%)	26 (5.2%)	9 (1.8%)	1 (0.2%)
4. I believe my likelihood of developing venous thromboembolism is low. (N)	26 (5.2%)	39 (7.7%)	111 (22%)	237 (47%)	91 (18.1%)
5. I feel I have sufficient understanding of the risks associated with venous thrombosis. (P)	30 (6%)	36 (7.1%)	178 (35.3%)	213 (42.3%)	47 (9.3%)
6. I believe taking preventive measures can significantly reduce my risk of venous thrombosis. (P)	302 (59.9%)	142 (28.2%)	53 (10.5%)	5 (1%)	2 (0.4%)
7. I am concerned about the costs of treatment for venous thromboembolism. (N)	296 (58.7%)	150 (29.8%)	44 (8.7%)	14 (2.8%)	0 (0%)
8. I feel worried about my risk of developing venous thrombosis. (N)	308 (61.1%)	131 (26%)	45 (8.9%)	19 (3.8%)	1 (0.2%)
9. I feel tired of undergoing treatment. (N)	321 (63.7%)	117 (23.2%)	40 (7.9%)	23 (4.6%)	3 (0.6%)
10. The prevention of venous thromboembolism makes me feel exhausted. (N)	305 (60.5%)	132 (26.2%)	33 (6.5%)	32 (6.3%)	2 (0.4%)
11. I believe that venous thromboembolism is primarily the physician's responsibility. (N)	20 (4%)	9 (1.8%)	12 (2.4%)	280 (55.6%)	183 (36.3%)
12. I am willing to follow my doctor's recommendations and take necessary measures to prevent venous thrombosis. (P)	329 (65.3%)	141 (28%)	25 (5%)	6 (1.2%)	3 (0.6%)
13. I have confidence in my doctor's treatment plan. (P)	266 (52.8%)	205 (40.7%)	33 (6.5%)	0 (0%)	0 (0%)
14. I can understand adjusting the treatment plan due to vascular thrombosis. (P)	270(53.6%)	198(39.3%)	34(6.7%)	0 (0%)	2(0.4%)

**Table 2.3:** Distribution of responses to practice dimension questions regarding VTE-related behaviors.

Practice	Strongly agree	Agree	Neutral	Disagree	Strongly disagree
1. I actively seek knowledge related to venous thromboembolism. (P)	262 (52%)	164 (32.5%)	55 (10.9%)	19 (3.8%)	4 (0.8%)
2. I try to increase my physical activity as much as possible. (P)	252 (50%)	177 (35.1%)	49 (9.7%)	18 (3.6%)	8 (1.6%)
3. I actively communicate with my doctor about the side effects of treatments. (P)	264 (52.4%)	197 (39.1%)	42 (8.3%)	1 (0.2%)	0 (0%)
4. I follow my doctor's recommendations and undergo regular VTE risk assessments. (P)	257 (51%)	216 (42.9%)	26 (5.2%)	5 (1%)	0 (0%)
5. I seek help from my doctor and address symptoms or issues related to venous thrombosis in a timely manner. (P)	243 (48.2%)	217 (43.1%)	40 (7.9%)	4 (0.8%)	0 (0%)
6. When sitting or bedridden for long periods, I take preventive measures against venous thrombosis, such as moving my legs or changing positions. (P)	260 (51.6%)	196 (38.9%)	46 (9.1%)	2 (0.4%)	0 (0%)
7. I maintain good lifestyle habits, such as quitting smoking and limiting alcohol consumption, to reduce the risk of venous thrombosis. (P)	296 (58.7%)	173 (34.3%)	32 (6.3%)	2 (0.4%)	1 (0.2%)
8. I share my experiences and knowledge about VTE risk management with other patients who have multiple myeloma (P)	273 (54.2%)	155 (30.8%)	52 (10.3%)	20 (4%)	4 (0.8%)
9. When I feel anxious about the disease, I talk to my family about it. (P)	284 (56.3%)	174 (34.5%)	36 (7.1%)	7 (1.4%)	3 (0.6%)
10. I express my concerns about the treatment plan to my doctor. (P)	302 (59.9%)	147(29.2%)	44(8.7%)	11(2.2%)	0 (0%)

### Correlations between KAP

Correlation analysis revealed significant positive relationships between knowledge and attitude (*r* = 0.141, *P* = 0.002) and between knowledge and practice (*r* = 0.281, *P* < 0.001). A significant correlation was also observed between attitude and practice (*r* = 0.159, *P* < 0.001) (Table 3).

**Table 3:** Spearman correlation coefficients between knowledge, attitude, and practice dimensions.

Spearman	Knowledge	Attitude	Practice
Knowledge	1.000		
Attitude	−0.141 (*P* = 0.002)	1.000	
Practice	0.281 (*P* < 0.001)	−0.159 (*P* < 0.001)	1.000

### Univariate and multivariate analysis of knowledge, attitude, and practice dimensions

The median of the knowledge, attitude, and practice scores were used as the cut-off value for each dimension to divide the groups, and the number of participants above the cut-off value were 340 (67.46%), 322 (63.89%), and 253 (50.20%), respectively (Table 4). Multivariate logistic regression showed that with monthly income of 2,000–5,000 Yuan [OR = 3.203, 95% CI: (1.205, 8.513), *P* = 0.020], with both social and commercial medical insurance [OR = 2.703, 95% CI: (1.019, 7.171), *P* = 0.046], not being informed by doctors about risk of venous thromboembolism [OR = 0.087, 95% CI: (0.016, 0.485), *P* = 0.005], with high risk on Padua Score [OR = 0.313, 95% CI: (0.124, 0.787), *P* = 0.014], and with medium risk on IMPEDEVTE Score [OR = 1.766, 95% CI: (1.104, 2.825), *P* = 0.018] were independently associated with knowledge (Table 4.1). Meanwhile, full-time homemaker [OR = 0.374, 95% CI: (0.183, 0.762), *P* = 0.007], with monthly income of 2,000–5,000 Yuan [OR = 2.968, 95% CI: (1.140, 7.727), *P* = 0.026], with monthly income greater than 5,000 yuan [OR = 4.094, 95% CI: (1.072, 15.637), *P* = 0.039], and with venous thromboembolism [OR = 0.574, 95% CI: (0.376, 0.878), *P* = 0.010] were independently associated with attitude (Table 4.2). Furthermore, with monthly income of 2,000–5,000 Yuan [OR = 3.326, 95% CI: (1.056, 10.480), *P* = 0.040], with high risk on Padua Score [OR = 0.185, 95% CI: (0.062, 0.553), *P* = 0.002], and with high risk on IMPEDEVTE Score [OR = 5.270, 95% CI: (1.024, 27.115), *P* = 0.047] were independently associated with practice (Table 4.3).

**Table 4:** Cutoff values for KAP dimensions based on median scores for logistic regression analysis.

Cutoff value: median	*N* (%)
Knowledge dimension total score	
Ksum > =9	340 (67.46%)
Ksum < =8	164 (32.54%)
Attitude dimension total score	
Asum > =29	322 (63.89%)
Asum < =28	182 (36.11%)
Practice dimension total score	
Psum > =45	253 (50.20%)
Psum < =44	251 (49.80%)

**Table 4.1:** Univariate and multivariate analysis for knowledge dimension.

Knowledge	Univariate analysis		Multivariate analysis	
	OR(95% CI)	*P*	OR(95% CI)	*P*
Gender				
Male				
Female	0.768 (0.522, 1.132)	0.180		
Age				
Under 60 years old				
60–69 years old	0.648 (0.376, 1.096)	0.110	0.720 (0.374, 1.387)	0.326
70 years old and above	0.598 (0.352, 0.994)	0.051	0.666 (0.343, 1.292)	0.229
BMI				
<18.5	1.134 (0.515, 2.687)	0.763		
18.49–23.99				
>=24.0	1.268 (0.799, 2.049)	0.323		
Ethnicity				
Han				
Minority	1.963 (0.614, 8.706)	0.301		
Residence				
Rural/suburban				
Urban	1.812 (1.237, 2.675)	0.003	1.392 (0.826, 2.347)	0.215
Education				
Middle school or below				
High school/technical school	1.662 (1.125, 2.463)	0.011	0.849 (0.500, 1.441)	0.544
Associate degree/bachelor's degree	3.037 (1.465, 6.950)	0.005	1.001 (0.298, 3.358)	0.999
Employment status				
Full-time				
Part-time/self-employed/freelancer	0.697 (0.399, 1.188)	0.192	0.844 (0.448, 1.589)	0.599
Unemployed/laid off	0.094 (0.041, 0.203)	<0.001	0.502 (0.178, 1.420)	0.194
Full-time homemaker	0.684 (0.358, 1.302)	0.248	1.030 (0.476, 2.229)	0.940
Retired	0.502 (0.241, 1.052)	0.066	1.076 (0.435, 2.663)	0.874
Monthly income				
<2,000				
2,000–5,000	6.744 (3.074, 16.391)	<0.001	3.203 (1.205, 8.513)	0.020
>5,000	14.143 (4.765, 47.660)	<0.001	4.107 (0.924, 18.257)	0.063
Marital status				
Married				
Divorced	0.427 (0.208, 0.872)	0.019	0.460 (0.203, 1.040)	0.062
Type of health insurance				
Social medical insurance only				
Both social and commercial medical insurance	2.796 (1.442, 5.977)	0.004	2.703 (1.019, 7.171)	0.046
No insurance	0.345 (0.114, 0.976)	0.048	0.538 (0.162, 1.781)	0.310
Whether the doctor informed you about the risk of venous thromboembolism				
No	0.038 (0.006, 0.132)	<0.001	0.087 (0.016, 0.485)	0.005
Yes				
Venous thromboembolism				
No				
Yes	1.234 (0.846, 1.796)	0.274		
Type of multiple myeloma				
IgA myeloma				
IgG myeloma	1.226 (0.792, 1.887)	0.357		
Light chain myeloma	1.153 (0.596, 2.284)	0.676		
Other (b/d/e/g/h)	1.683 (0.475, 7.866)	0.452		
Multiple Myeloma Staging (R-ISS)				
Stage I				
Stage II	1.266 (0.757, 2.096)	0.363		
Stage III	1.253 (0.703, 2.226)	0.441		
Padua Score				
Low risk = 0–3 points				
High risk ≥ 4 points	0.127 (0.064, 0.241)	<0.001	0.313 (0.124, 0.787)	0.014
Khorana Score				
Low risk: total score 0 point				
Medium risk: total socre 1–2 points	2.355 (1.488, 3.728)	<0.001	1.162 (0.626, 2.155)	0.634
High risk: total score > 3 points	1.565 (0.537, 4.920)	0.421	1.457 (0.285, 7.450)	0.651
IMPEDEVTE Score				
Low risk: total score ≤ 3 points				
Medium risk: total score 4–7 points	1.965 (1.335, 2.897)	0.001	1.766 (1.104, 2.825)	0.018
High risk: total score ≥ 8 points	0.431 (0.126, 1.342)	0.153	0.781 (0.145, 4.204)	0.774

**Table 4.2:** Univariate and multivariate analysis for attitude dimension.

Attitude	Univariate analysis		Multivariate analysis	
	OR (95% CI)	*P*	OR (95% CI)	*P*
Knowledge	0.947 (0.887, 1.010)	0.099	0.959 (0.874, 1.053)	0.378
Gender				
Male				
Female	0.757 (0.519, 1.106)	0.148		
Age				
Under 60 years old				
60–69 years old	1.044 (0.633, 1.712)	0.867		
70 years old and above	0.933 (0.575, 1.503)	0.778		
BMI				
<18.5	1.185 (0.547, 2.723)	0.675	1.156 (0.495, 2.701)	0.738
18.49–23.99				
>=24.0	1.550 (0.980, 2.499)	0.066	1.352 (0.797, 2.293)	0.263
Ethnicity				
Han				
Minority	0.844 (0.299, 2.553)	0.751		
Residence				
Rural/suburban				
Urban	1.467 (1.015, 2.129)	0.043	1.311 (0.806, 2.133)	0.276
Education				
Middle school or below				
High school/technical school	0.986 (0.674, 1.442)	0.941	0.760 (0.467, 1.238)	0.271
Associate degree/bachelor's degree	1.882 (0.955, 3.947)	0.079	0.679 (0.256, 1.800)	0.436
Employment status				
Full-time				
Part-time/self-employed/freelancer	0.565 (0.334, 0.935)	0.029	0.651 (0.364, 1.163)	0.147
Unemployed/laid off	1.314 (0.593, 3.093)	0.514	1.271 (0.422, 3.827)	0.670
Full-time homemaker	0.338 (0.184, 0.611)	<0.001	0.374 (0.183, 0.762)	0.007
Retired	0.519 (0.255, 1.060)	0.070	0.484 (0.219, 1.066)	0.072
Monthly income				
<2,000				
2,000–5,000	1.530 (0.736, 3.153)	0.248	2.968 (1.140, 7.727)	0.026
>5,000	3.039 (1.119, 8.674)	0.032	4.094 (1.072, 15.637)	0.039
Marital status				
Married				
Divorced	0.659 (0.324, 1.359)	0.251		
Type of health insurance				
Social medical insurance only				
Both social and commercial medical insurance	1.388 (0.795, 2.507)	0.261		
No insurance	0.879 (0.311, 2.665)	0.810		
Whether the doctor informed you about the risk of venous thromboembolism				
No	2.947 (1.094, 10.254)	0.052	0.783 (0.189, 3.245)	0.736
Yes				
Venous thromboembolism				
No				
Yes	0.467 (0.317, 0.683)	<0.001	0.574 (0.376, 0.878)	0.010
Type of multiple myeloma				
IgA myeloma				
IgG myeloma	1.243 (0.814, 1.892)	0.312	1.311 (0.833, 2.064)	0.243
Light chain myeloma	1.884 (0.960, 3.843)	0.072	1.889 (0.902, 3.952)	0.092
Other (b/d/e/g/h)	3.533 (0.886, 23.598)	0.112	3.695 (0.706, 19.328)	0.122
Multiple Myeloma Staging (R-ISS)				
Stage I				
Stage II	0.696 (0.406, 1.165)	0.176		
Stage III	0.742 (0.409, 1.326)	0.318		
Padua Score				
Low risk = 0–3 points				
High risk ≥ 4 points	2.963 (1.474, 6.626)	0.004	1.984 (0.761, 5.171)	0.161
Khorana Score				
Low risk: total score 0 point				
Medium risk: total socre 1–2 points	0.512 (0.302, 0.842)	0.010	0.835 (0.465, 1.500)	0.547
High risk: total score > 3 points	0.319 (0.106, 0.958)	0.040	0.556 (0.166, 1.859)	0.340
IMPEDEVTE Score				
Low risk: total score ≤ 3 points				
Medium risk: total score 4–7 points	0.947 (0.647, 1.381)	0.777		
High risk: total score ≥ 8 points	0.630 (0.202, 2.030)	0.424		

**Table 4.3:** Univariate and multivariate analysis for practice dimension.

Practice	Univariate analysis		Multivariate analysis	
	OR (95% CI)	*P*	OR (95% CI)	*P*
Knowledge	1.182 (1.104, 1.266)	<0.001	1.020 (0.931, 1.118)	0.666
Attitude	0.916 (0.857, 0.979)	0.010	0.973 (0.897, 1.057)	0.518
Gender				
Male				
Female	0.721 (0.498, 1.040)	0.081	0.914 (0.543, 1.536)	0.733
Age				
Under 60 years old				
60–69 years old	0.600 (0.371, 0.968)	0.037	0.651 (0.367, 1.155)	0.142
70 years old and above	0.817 (0.512, 1.298)	0.393	0.931 (0.514, 1.685)	0.812
BMI				
<18.5	0.636 (0.289, 1.360)	0.248		
18.49–23.99				
>=24.0	0.721 (0.467, 1.109)	0.138		
Ethnicity				
Han				
Minority	0.486 (0.150, 1.388)	0.194		
Residence				
Rural/suburban				
Urban	1.499 (1.054, 2.138)	0.025	1.167 (0.723, 1.885)	0.528
Education				
Middle school or below				
High school/technical school	1.739 (1.201, 2.527)	0.004	1.270 (0.778, 2.072)	0.339
Associate degree/bachelor's degree	2.270 (1.218, 4.327)	0.011	1.766 (0.640, 4.869)	0.272
Employment status				
Full-time				
Part-time/self-employed/freelancer	1.054 (0.658, 1.683)	0.825	1.167 (0.670, 2.032)	0.586
Unemployed/laid off	0.129 (0.050, 0.298)	<0.001	0.571 (0.194, 1.674)	0.307
Full-time homemaker	0.619 (0.351, 1.085)	0.095	0.861 (0.396, 1.873)	0.706
Retired	0.646 (0.327, 1.264)	0.203	0.780 (0.347, 1.755)	0.548
Monthly income				
<2,000				
2,000–5,000	5.924 (2.432, 17.731)	<0.001	3.326 (1.056, 10.480)	0.040
>5,000	6.600 (2.244, 22.677)	0.001	2.932 (0.668, 12.866)	0.154
Marital status				
Married				
Divorced	0.715 (0.344, 1.453)	0.357		
Type of health insurance				
Social medical insurance only				
Both social and commercial medical insurance	1.772 (1.039, 3.079)	0.038	1.414 (0.684, 2.923)	0.350
No insurance	0.930 (0.321, 2.636)	0.891	1.199 (0.370, 3.878)	0.762
Whether the doctor informed you about the risk of venous thromboembolism				
No	0.000 (0.000, 18,871.490)	0.973		
Yes				
Venous thromboembolism				
No				
Yes	1.136 (0.798, 1.619)	0.479		
Type of multiple myeloma				
IgA myeloma				
IgG myeloma	0.970 (0.642, 1.466)	0.886		
Light chain myeloma	0.842 (0.446, 1.586)	0.595		
Other (b/d/e/g/h)	0.470 (0.120, 1.570)	0.236		
Multiple Myeloma Staging (R-ISS)				
Stage I				
Stage II	1.078 (0.663, 1.757)	0.761		
Stage III	1.049 (0.607, 1.815)	0.865		
Padua Score				
Low risk = 0–3 points				
High risk ≥ 4 points	0.108 (0.041, 0.240)	<0.001	0.185 (0.062, 0.553)	0.002
Khorana Score				
Low risk: total score 0 point				
Medium risk: total socre 1–2 points	2.806 (1.752, 4.588)	<0.001	1.769 (0.987, 3.171)	0.055
High risk: total score > 3 points	1.770 (0.582, 5.214)	0.300	0.838 (0.202, 3.474)	0.807
IMPEDEVTE Score				
Low risk: total score ≤ 3 points				
Medium risk: total score 4–7 points	1.804 (1.252, 2.611)	0.002	1.464 (0.946, 2.266)	0.087
High risk: total score ≥ 8 points	1.215 (0.378, 3.792)	0.735	5.270 (1.024, 27.115)	0.047

### Interactions between KAP

**Figure 1:**
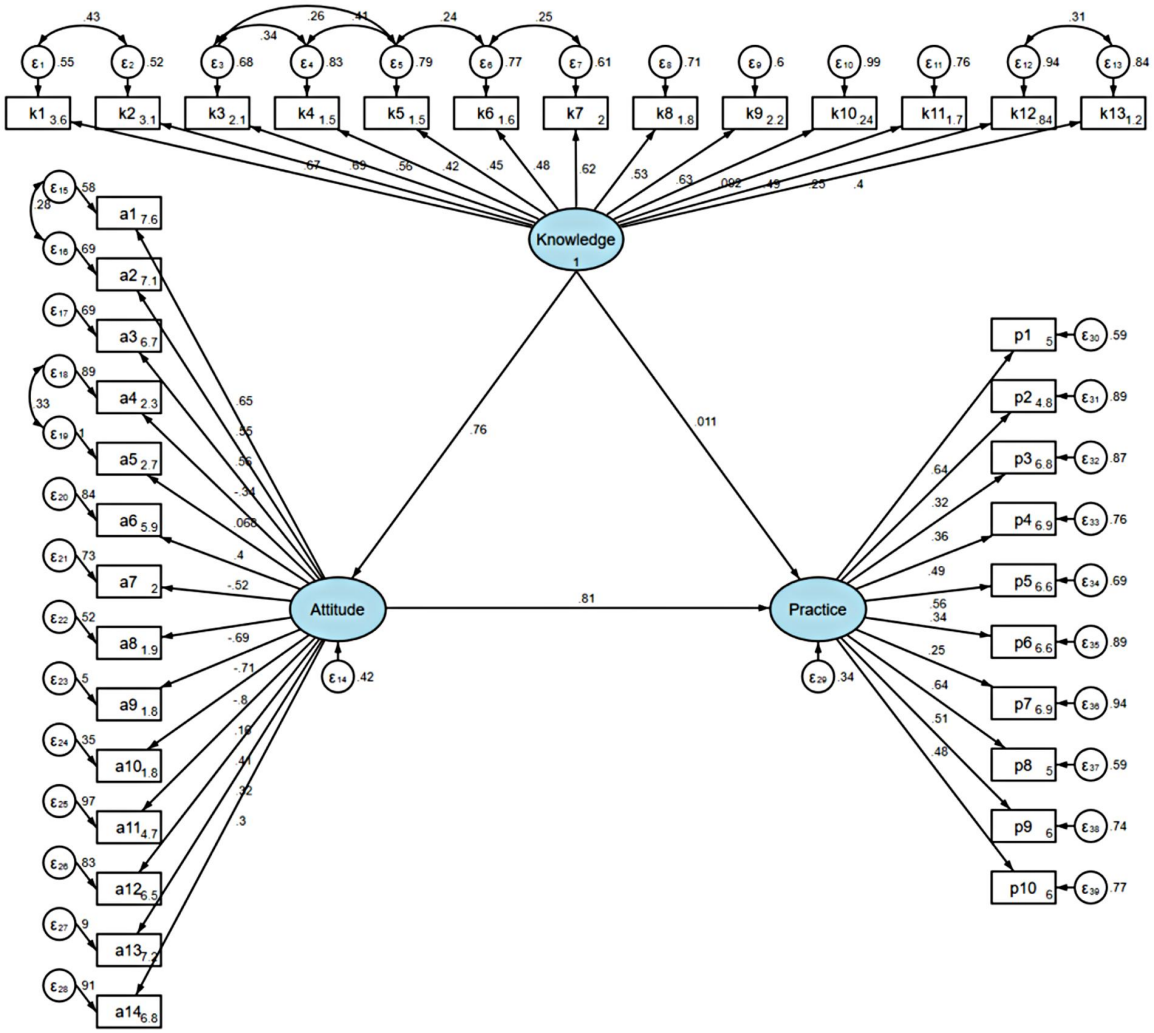
SEM model analysis results.

The SEM analysis demonstrated a good model fit (RMSEA = 0.046, SRMR = 0.055, TLI = 0.855, and CFI = 0.865) (Supplementary Table S2). The detailed effects among KAP dimensions are presented in SupplementaryTable S3. Direct and indirect effect analyses showed that knowledge had a direct effect on attitude (*β* = 0.761, *P* < 0.001), attitude directly influenced practice (*β* = 0.806, *P* < 0.001), and knowledge indirectly affected practice via attitude (*β* = 0.613, *P* < 0.001) (Supplementary Table S4 and Figure 1).

## Discussion

This study represents the first comprehensive assessment of KAP regarding VTE among Chinese MM patients, revealing significant knowledge deficits and negative attitudes despite generally proactive practice behaviors. When compared to international guidelines from ASCO, ESMO, and NCCN for VTE prevention in cancer patients (9, 21, 22), our findings highlight substantial gaps between recommended care standards and patient understanding. The mean knowledge score (8.97 ± 2.92 out of 13) corresponds to approximately 69% of the maximum possible score, which falls below the predefined 70% threshold used to indicate adequate knowledge. In clinical terms, this suggests that a substantial proportion of MM patients lack mastery of key VTE-related concepts, particularly regarding treatment duration and anticoagulation management. The 70% cutoff, commonly adopted in KAP studies of chronic diseases, is generally considered to reflect a minimally acceptable level of health literacy necessary to support informed decision-making and sustained preventive behavior. Compared with other chronic disease KAP studies, where adequate knowledge levels frequently exceed 75%–80% of the maximum score, the findings in our cohort indicate a relative deficit in VTE-specific understanding. This suggests that current patient education strategies may not sufficiently address the complex and treatment-dependent thrombotic risks associated with MM.

The findings are consistent with previous studies in other chronic disease populations, which similarly reported insufficient awareness and preventative behaviors related to VTE (12, 13). This insufficient KAP may explain why complications such as VTE recurrence or delayed detection continue to be prevalent among MM patients despite advances in treatment protocols. Notably, this study found that misconceptions about VTE prevention and treatment were common, particularly regarding the need for continuous preventive measures and the role of anticoagulant therapy. These gaps align with findings from prior research indicating that patients often underestimate the importance of sustained preventive care, especially when symptoms are not immediately apparent (23, 24). This further highlights the urgent need to address both informational and emotional barriers to improve adherence and outcomes.

The correlation analysis and SEM results demonstrated significant relationships among KAP dimensions, where knowledge directly influenced attitudes, which in turn significantly impacted practices. Knowledge also indirectly influenced practices through attitudes. These findings align with the KAP model, which posits that increased awareness leads to improved attitudes and, consequently, better practices (25, 26). Structural equation modeling is a multivariate statistical approach that allows simultaneous estimation of multiple direct and indirect relationships among observed and latent variables within a theoretically specified framework. Unlike simple regression models, SEM enables the testing of complex pathway structures and mediating effects while accounting for measurement error. In KAP research, SEM is frequently used to evaluate whether empirical data support the hypothesized knowledge → attitude → practice sequence and to quantify the relative strength of direct and indirect pathways. Although causal inference cannot be established in cross-sectional designs, SEM provides a structured method to assess the plausibility and internal consistency of theoretical models. Therefore, its application in the present study serves to statistically examine the coherence of the KAP framework rather than to claim causal determination. However, the relatively modest correlations suggest that other factors, such as psychological distress, social support, or systemic healthcare barriers, may moderate these effects. This is supported by other studies that report knowledge does not always translate into action, particularly when patients face emotional or practical obstacles (27, 28). The apparent contradiction between low knowledge scores and high practice scores may reflect social desirability bias, whereby patients report behaviors they perceive as expected rather than their actual practices. This phenomenon has been documented in chronic disease KAP studies and highlights the limitations of relying solely on self-reported behavioral measures. Beyond social desirability bias, this paradox also raises concerns regarding the construct validity of the practice dimension. Patients may have interpreted practice items as reflecting general compliance with medical advice rather than VTE-specific preventive behaviors. Moreover, high reported practice scores may represent passive adherence to physician-directed care rather than autonomous, knowledge-driven self-management. This distinction is clinically important, as effective prevention requires both accurate understanding and intentional behavioral engagement. Therefore, reported practice levels should be interpreted cautiously. It should be noted that data in this study were collected anonymously to reduce reporting pressure; however, anonymity alone cannot fully eliminate response distortion. Future research could strengthen behavioral assessment by triangulating self-reported practices with clinical indicators, such as documented adherence to anticoagulation therapy or attendance at scheduled risk assessments, and by incorporating longitudinal or mixed-method designs to better evaluate consistency between reported and actual preventive behaviors.

Key demographic and clinical variables were associated with significant differences in KAP scores. For example, patients with higher monthly income and those informed by doctors about VTE risks consistently demonstrated better KAP outcomes. Multivariate analysis confirmed that income (2,000–5,000 Yuan) and being informed by a doctor were strong predictors of higher knowledge and practice scores. These findings align with research indicating that socioeconomic status and clinician-patient communication play critical roles in shaping health literacy and adherence (29, 30).

On the other hand, rural residents, patients with lower education levels, and those without comprehensive health insurance had significantly poorer KAP outcomes. This highlights persistent disparities in healthcare access and education, which are well-documented in other studies on chronic disease management (31). The finding that education level did not significantly impact attitudes suggests that negative perceptions, such as the financial burden of VTE treatment, may be more influenced by experiential and contextual factors than by formal education. Similarly, rural residents showed lower scores across all three dimensions, likely due to limited access to healthcare resources and educational opportunities.

These disparities underscore important health equity implications. Patients residing in rural or underserved areas may encounter structural barriers such as reduced access to hematology specialists, fewer educational resources, transportation limitations, and greater financial constraints. Such systemic disadvantages may compound the complex management demands of MM and widen disparities in VTE-related knowledge and preventive engagement. Addressing these inequities requires targeted and scalable strategies. Telehealth-based education programs could facilitate remote risk communication and follow-up assessment, particularly for geographically isolated patients. Community health workers or trained primary care providers may serve as accessible channels for delivering standardized, culturally appropriate VTE prevention education. In addition, mobile health tools, including SMS reminder systems or simplified digital applications, could reinforce preventive behaviors and improve adherence. Integrating VTE education into broader rural health initiatives may help ensure that prevention strategies are equitable, sustainable, and accessible to vulnerable populations.

Notably, patients classified as high-risk on the Padua and IMPEDEVTE scores had significantly poorer KAP outcomes. Multivariate analysis confirmed that these high-risk groups were independently associated with lower knowledge and practice scores. This may reflect both the clinical complexity of managing high-risk cases and the emotional burden associated with increased awareness of one's health risks.

The distribution of responses in the knowledge dimension revealed several areas of concern, such as a lack of understanding about the duration of VTE prevention and misconceptions about anticoagulant therapy. Similar findings have been reported in other studies, where patients often struggle to grasp the chronic nature of VTE management (32, 33). This suggests that educational efforts must address not only the “what” but also the “why” of prevention strategies to ensure deeper comprehension. In addition to these knowledge gaps, it is noteworthy that 57.7% of participants in our cohort reported a prior history of VTE. This prevalence appears higher than that reported in many MM cohorts. The elevated proportion may be partly attributable to the broad inclusion of any documented venous thrombotic event, including catheter-related thrombosis and remote historical events occurring either before or after MM diagnosis. Because all confirmed VTE events were recorded regardless of timing, the cumulative lifetime prevalence within this tertiary-care cohort may have been captured rather than incident treatment-related events alone. Furthermore, although VTE history was verified through medical records when available, some degree of recall inaccuracy or event misclassification cannot be completely excluded. Importantly, prior VTE experience may have influenced KAP outcomes in two opposing directions: it may enhance awareness and engagement while simultaneously contributing to anxiety or negative attitudes toward prevention and treatment burden. Therefore, this characteristic of the study population should be considered when interpreting the magnitude and generalizability of the findings.

In the attitude dimension, while most patients valued the importance of prevention and expressed confidence in their doctors, many also reported feeling burdened by preventive measures and concerned about treatment costs. This duality mirrors findings from other studies where financial and emotional stress were shown to hinder adherence to chronic disease management plans (34, 35). Specific focus on reducing this burden through financial support or streamlined care protocols could alleviate these barriers.

In the practice dimension, while the majority of patients reported engaging in proactive behaviors, such as seeking knowledge and communicating with doctors, notable gaps remained. For instance, a significant proportion of patients were neutral or inactive in sharing knowledge with peers or maintaining healthy lifestyle habits. This lack of community engagement is a missed opportunity, as peer support has been shown to significantly enhance health behaviors in similar populations (36, 37).

To address the deficiencies observed in this study, a multifaceted approach targeting both systemic and individual factors is necessary. First, evidence-based educational interventions similar to those successfully implemented in other cancer populations should be adapted for MM patients. These should specifically target the knowledge gaps identified in our study, including misconceptions about treatment duration (36.3% uncertainty) and anticoagulation management during active treatment (64.3% incorrect responses). These programs should use culturally tailored, interactive materials to ensure accessibility for patients with varying literacy levels. For rural residents, where healthcare access is limited, telehealth initiatives and mobile applications could serve as effective platforms for delivering educational content. Additionally, healthcare providers should be trained to communicate VTE risks and prevention strategies effectively, emphasizing the importance of tailoring messages to patients' individual circumstances. Providing patients with written or digital summaries of consultations could reinforce their understanding and facilitate long-term retention of critical information (21, 22).

Psychosocial support is also vital in mitigating the emotional and financial burdens identified in the attitude dimension. Counseling services, either individually or in group settings, could help patients manage anxiety related to VTE prevention and treatment. These sessions could also promote peer interaction, enabling patients to share experiences and develop coping strategies. Financial concerns, a significant barrier to positive attitudes, could be alleviated by collaborating with insurance providers to subsidize preventive measures and treatment costs. For patients in rural or lower-income settings, these subsidies could make preventive care more accessible and sustainable (38, 39).

Moreover, the gaps observed in the practice dimension highlight the need for interventions that promote actionable behaviors. Encouraging patients to maintain healthy lifestyle habits, such as increased physical activity and smoking cessation, could be achieved through targeted campaigns. Providing patients with wearable devices or activity trackers could further enhance engagement by offering real-time feedback on their efforts. To promote community engagement, establishing peer support groups could serve as an effective strategy for fostering shared learning and accountability. These groups can also be platforms for disseminating educational materials and reinforcing the importance of preventive practices. Lastly, implementing follow-up systems could significantly improve adherence to preventive measures. Regular reminders via phone calls, SMS, or mobile apps can prompt patients to attend risk assessments, communicate with healthcare providers, and maintain healthy behaviors. For high-risk patients, more intensive monitoring may be necessary to ensure consistent engagement (40, 41).

Based on our findings, clinicians should implement systematic VTE risk communication protocols that go beyond current practice standards. Specifically, healthcare providers should: (1) conduct structured knowledge assessments using validated tools to identify individual patient gaps; (2) provide written educational materials that address the most common misconceptions identified in this study, particularly regarding treatment duration and anticoagulation management; (3) implement shared decision-making approaches that acknowledge patient concerns about treatment burden and costs; and (4) establish regular follow-up protocols to reinforce education and assess adherence to preventive measures. These interventions should be tailored to address the socioeconomic and educational disparities identified in our analysis.

This study has several limitations. First, as a cross-sectional survey, it assessed participants at a single time point, limiting the ability to evaluate changes in KAP over time or infer causality. Second, reliance on self-reported data introduces potential recall and social desirability bias, which may have affected response accuracy. Third, participants were recruited through convenience sampling from a single tertiary hospital, limiting external validity. Patients treated at tertiary centers may have greater access to specialized care, more frequent physician interaction, and higher health literacy than those in community or rural settings; thus, the observed KAP levels may not fully represent the broader MM population. Multi-center or population-based studies may yield different KAP distributions due to variations in socioeconomic background, healthcare access, institutional practices, and educational resources. Therefore, the findings should be interpreted with caution, and geographically diverse studies are needed to enhance generalizability. In addition, caregiver perspectives were not assessed. Because many MM patients, particularly older individuals, rely on family members for treatment decisions and daily management, the absence of caregiver input may limit a full understanding of preventive behaviors. Although VTE history was verified through medical records when available, recall bias may still have influenced certain self-reported information. Finally, objective adherence indicators, such as pharmacy refill records, anticoagulation monitoring data, or wearable activity tracking, were not included, which may have led to overestimation of actual preventive engagement. Future studies incorporating caregiver perspectives and objective behavioral measures would strengthen the validity of KAP findings in MM populations.

## Conclusions

In conclusion, patients with MM demonstrated suboptimal knowledge, negative attitudes, and proactive practices regarding VTE. Correlation and structural equation modeling analyses highlighted that knowledge significantly influences attitude, which in turn impacts practice, underscoring the interconnected nature of these dimensions. These findings suggest that targeted educational interventions to improve knowledge about VTE could positively influence attitudes and enhance proactive practices, ultimately supporting better prevention and management outcomes in clinical settings.

## Data Availability

The original contributions presented in the study are included in the article/Supplementary Material, further inquiries can be directed to the corresponding author.

## Supplementary material

Supplementary Figure S1 CFA model.
