# Supplementary material for: Knowledge, attitudes, and practices of patients with multiple myeloma regarding venous thromboembolism: a cross-sectional study
Source: Front Cardiovasc Med. 2026 Apr 24;13:1722955. doi: 10.3389/fcvm.2026.1722955 (PMC13154387; doi:10.3389/fcvm.2026.1722955)
Supplement: Supplementary file 1 [file Datasheet1.docx]

**Supplementary table 1. CFA fit**

| **Indicators** | **Reference** | **Results** |
| --- | --- | --- |
| RMSEA | <0.08Good | 0.046 |
| SRMR | <0.08Good | 0.055 |
| TLI | >0.8Good | 0.855 |
| CFI | >0.8Good | 0.865 |

**Supplementary table 2. SEM model fit**

| **Indicators** | **Reference** | **Results** |
| --- | --- | --- |
| RMSEA | <0.08Good | 0.046 |
| SRMR | <0.08Good | 0.055 |
| TLI | >0.8Good | 0.855 |
| CFI | >0.8Good | 0.865 |

**Supplementary table 3. SEM model path analysis results**

| **Indicators** |  | **Estimate** | **P>\|z\|** |
| --- | --- | --- | --- |
| Asum |  |  |  |
|  | Ksum | 26.17 | <0.001 |
| Psum |  |  |  |
|  | Asum | 10.89 | <0.001 |
|  | Ksum | 14.93 | <0.001 |

**Supplementary table 4. Results of the analysis of the direct and indirect effects of the SEM model**

| **Model paths** | | Total effects | | Direct Effect | | Indirect effect | |
| --- | --- | --- | --- | --- | --- | --- | --- |
|  |  | β(95%CI) | P | β(95%CI) | P | β(95%CI) | P |
| Asum |  |  |  |  |  |  |  |
|  | Ksum | 0.761 (0.704,0.818) | <0.001 | 0.761 (0.704,0.818) | <0.001 |  |  |
| Psum |  |  |  |  |  |  |  |
|  | Asum | 0.806 (0.661,0.951) | <0.001 | 0.806 (0.661,0.951) | <0.001 |  |  |
|  | Ksum | 0.624 (0.542,0.706) | <0.001 | 0.011 (-0.152,0.174) | 0.896 | 0.613 (0.484,0.742) | <0.001 |
