# Supplementary figures and images for: Knowledge, attitudes, and practices of patients with multiple myeloma regarding venous thromboembolism: a cross-sectional study
Source: Front Cardiovasc Med. 2026 Apr 24;13:1722955. doi: 10.3389/fcvm.2026.1722955 (PMC13154387; doi:10.3389/fcvm.2026.1722955)

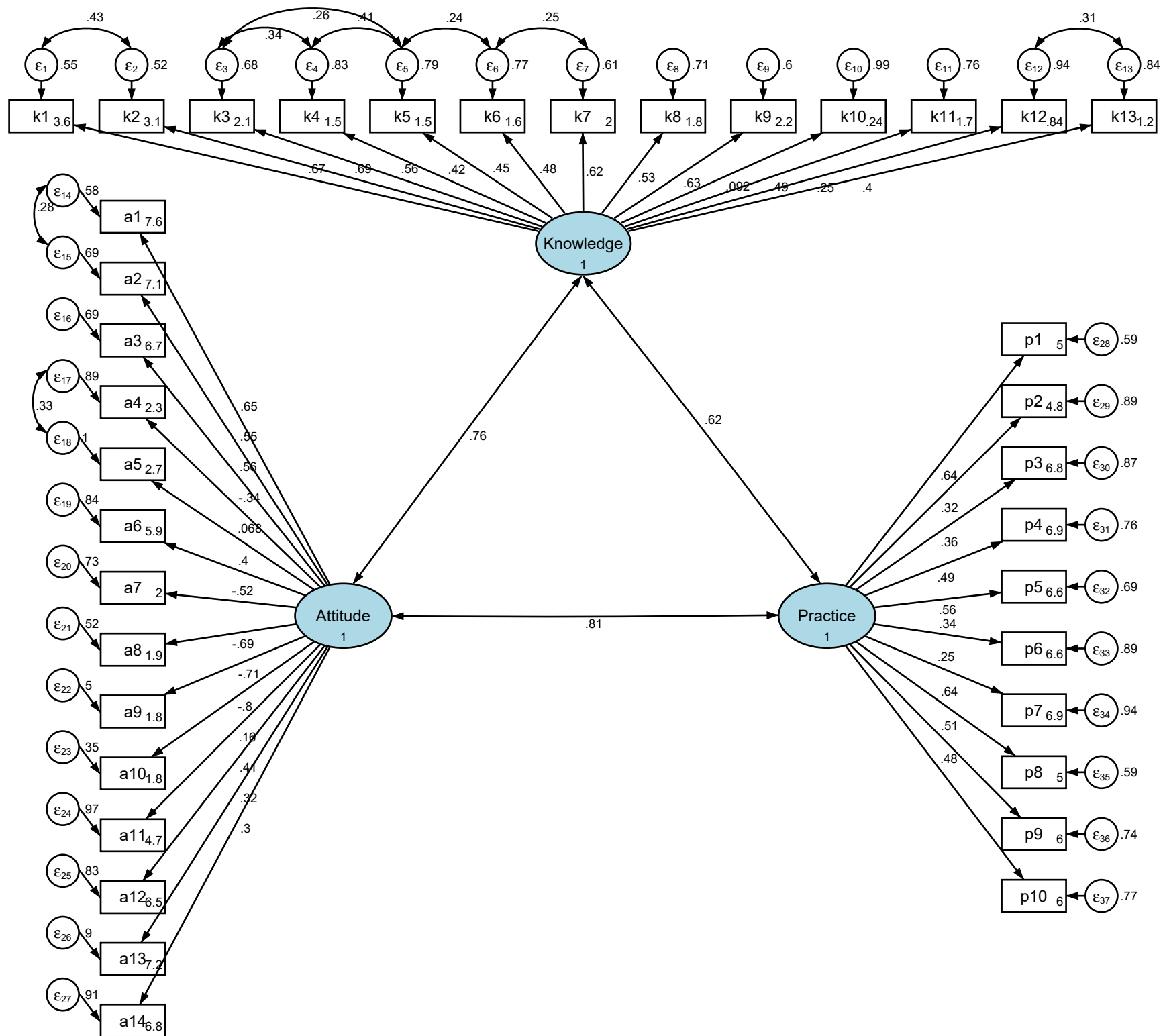

Supplement: Supplementary Figure S1 — CFA model. [file Image1.pdf]
